# Barriers to Access of Healthcare Services for Rural Women—Applying Gender Lens on TB in a Rural District of Sindh, Pakistan

**DOI:** 10.3390/ijerph181910102

**Published:** 2021-09-26

**Authors:** Shifa Salman Habib, Wafa Zehra Jamal, Syed Mohammad Asad Zaidi, Junaid-Ur-Rehman Siddiqui, Hira Mustafa Khan, Jacob Creswell, Srichand Batra, Anna Versfeld

**Affiliations:** 1 Community Health Solutions, Karachi 74000, Pakistan; shifa.habib@chshealthcare.org (S.S.H.); asad.zaidi@chshealthcare.org (S.M.A.Z.); 2 Aahung, Karachi 75600, Pakistan; Junaidrehman1994@hotmail.com (J.-U.-R.S.); hira.khan@aahung.org (H.M.K.); 3 The Stop TB Partnership, Chemin du Pommier 40, 1218 Le Grand-Saconnex, Switzerland; jacobc@stoptb.org (J.C.); annaversfeld@gmail.com (A.V.); 4 Pakistan Tuberculosis Program, Karachi 12000, Pakistan; doctor_srichand123@hotmail.com

**Keywords:** tuberculosis, patriarchy, gender barriers, women

## Abstract

Background: Women in the rural districts of Pakistan face numerous barriers to healthcare, rendering gender-responsive health programming important, including for the disease of tuberculosis (TB). This study was conducted to assess the general understanding of TB and for women’s access to healthcare, as a first step towards implementation of a gender responsive TB program in Tando Allahyar, a rural district of Pakistan. Methods: A total of 36 participants were interviewed for the study. The focus group discussion guide comprised of questions on: (1) family/household dynamics, (2) community norms, (3) healthcare systems, (4) women’s access to healthcare, (5) TB Awareness, and (6) women’s access to TB Care. Results: Limited autonomy in household financial decision-making, disapproval of unassisted travel, long travel time, lack of prioritization of spending on women’s health and inadequate presence of female health providers, were identified as barriers to access healthcare for women, which is even higher in younger women. Facilitators to access of TB care included a reported lack of TB-related stigma, moderate knowledge about TB disease, and broad understanding of tuberculosis as a curable disease. Other suggested facilitators include health facilities closer to the villages and the availability of higher quality services. Conclusion: Significant barriers are faced by women in accessing TB care in rural districts of Pakistan. Program implementers in high burden countries should shift towards improved gender-responsive TB programming.

## 1. Introduction

Gender is an important determinant of health, particularly in low-and-middle income countries (LMICs) [1,2] The importance of addressing gender inequalities in access to health care is well established in literature. Previous studies from multiple LMICs in African and South Asian regions have reported women to have experienced greater barriers to healthcare compared to men, resulting in overall poorer health outcomes and higher mortality [3,4,5,6,7]. A recent study from Pakistan reports lower uptake of community-based TB screening services among women despite a higher age standardized prevalence of active TB [8]. Pakistani society is highly patriarchal, and gender-based disparities are common between women and men in health, education, income opportunities, employment opportunities, personal security, control over assets and participation in politics [9,10] Pakistan ranks 151st on the World Economic Forum’s Global Gender Gap Report, third from last on the list [11]. Women face numerous, well-documented barriers to healthcare, including limited decision-making powers [12,13], household care responsibilities, restrictions on travelling alone, and the prioritization of male family members’ health [14] This context renders gender-responsive health programming particularly important. Yet, there is a dearth of initiatives that explore the barriers that women have to tuberculosis care.

Globally, tuberculosis (TB) is one of the top six causes of mortality among women aged 15–49 years of age [15]. Pakistan has the fifth highest burden of TB in the world and an estimated 42% of cases remain undiagnosed [16]. In line with global patterns, men experience a higher burden of TB disease than women in Pakistan. However, the cumulative reported male-female (M:F) ratio for TB notifications, at 1.1, [17] is one of the lowest in the world, indicating a proportionally high burden of TB in women. (Globally males have twice the TB case notifications compared to females [18]). There are also wide discrepancies in the M:F ratios between provinces and regions in Pakistan, suggesting the impact of socio-cultural and economic factors on TB infection and its notifications [19]. Women of reproductive age (15–34 years) also have a marginally higher burden of TB than men in the same age category [20].

The Stop TB Partnership’s TB REACH Wave 7 funding, supported by Global Affairs Canada, provided funding to projects that integrated a women’s empowerment approach into their TB programming. With this support, Community Health Solutions (CHS), a social enterprise based in Pakistan, implemented a project in Tando Allayar, a rural district of the Sindh Province. The Sindh Province has the country’s highest TB prevalence rate (454 per 100,000) and over two thirds 67.3% of the population people live under the poverty line [21]. Almost half of children aged under five are reportedly stunted or short for their age and a quarter are severely stunted [22].

According to unpublished National Tuberculosis Program (NTP) surveillance data, district Tando Allayar has one of the lowest recorded case detection rates in Pakistan (29%) and females account for 46% of case notification. Health services are limited. Only 29.1% of ever married women in Tando Allahyar complete four or more antenatal care visits [23]. The population of 575,720 is served by limited and poorly resourced health facilities including 14 Basic Health Units, three Rural Health Centers and one 100-bed tertiary-care hospital [24]. There are two TB Basic Management Units for TB in the public sector, marked by frequent staff-absenteeism and limited work hours. Hyderabad (40 km) and Kotri (50 km) are the closest large cities with public and private hospitals and facilities for TB care.

Noting the role that TB programming has in shifting harmful gender norms [19], TB REACH Wave 7 funded projects were required to incorporate aspects of women’s empowerment. Supported by this wave of funding, CHS developed a Rural Tele-Healthcare Hub at Tando Allahyar district. The Hub offered high quality TB diagnostics and treatment services to patients, including the use of digital chest x-rays and a microbiology laboratory, and notified patients to the National TB Program. The Hub also linked under-employed female doctors in major cities to rural women via tele-medicine, supported by Sehat Kahani, a private technology startup providing telehealth solutions [25]. Onsite, a Lady Health Visitor (LHV) provided maternal and child health services and initiated treatment for people with TB, under supervision of the online physicians. The Hub was linked with direct outreach for TB awareness, screening and treatment into communities through an existing network of over 200 women volunteers, through a pioneering approach for community-mobilization developed by National Rural Support Programme (NRSP) [26]. Under this approach, rural women are encouraged to advocate for their rights through village-based community organizations and participate in the implementation of development initiatives. Our project is the first adaptation of this approach for TB. Through these female volunteers, households are mapped, screened and persons with presumptive TB are identified and linked to the hub with a travel voucher.

To validate the design and ensure commensurate implementation of a gender-responsive TB program, the first step was the implementation of an assessment of general understanding of TB and of access to healthcare for women at the intervention site. This paper outlines the findings of this study and provides useful insights into key considerations for gender-responsive care in rural settings of Pakistan.

## 2. Methods

### 2.1. Study Design

**Figure 1:**
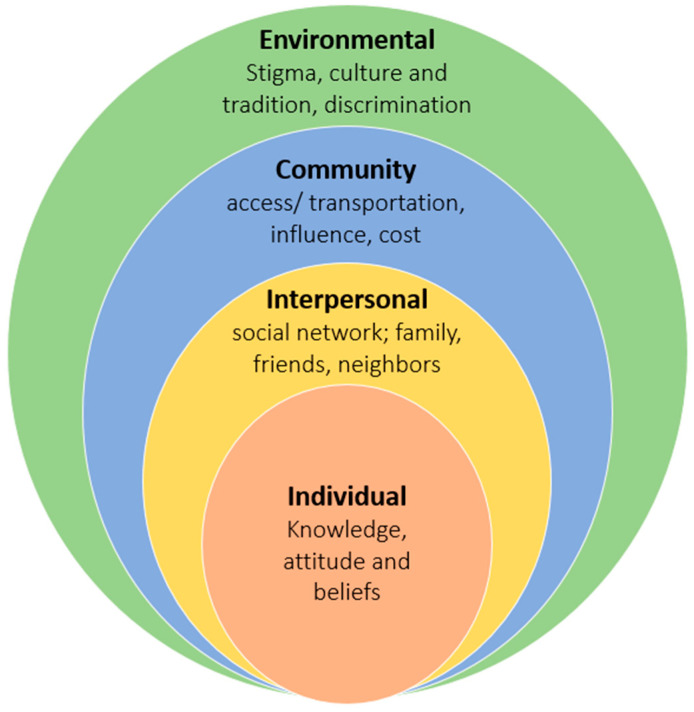
An adaptation of the socioecological model by McLeroy et al.

This study reports on exploratory focus group discussions (FGD), that were conducted using semi-structured interview guides, to understand the barriers and facilitators of access to general healthcare and TB services. Conceptually, we have drawn on the socioecological model (SEM) in the design and analysis phases for this study [27]. While the SEM identifies five nested, or hierarchical levels of influence, for our study we adapted the model to focus on the following four levels: individual, interpersonal, environmental/structural and community (Figure 1).

### 2.2. Participants

Women from three groups were included: younger lay community women aged 18 to 25 years; older lay community women aged 26 to 49 years; and community resource persons (CRPs) from the National Rural Support Programme (NRSP), the largest rural upliftment program in Pakistan working with 3.6 million poor households. CRPs are rural women who are recruited into community organizations and provided with trainings on leadership, communication, and community support. They engage in a range of development initiatives, including healthcare provision [28]. The inclusion of CRPs and lay women was to allow for the understanding of the differences in perspectives that may arise from differential exposure and training. The lay community were divided into age-group categories to encourage participation among peer groups and to investigate the presence of age-related differences in barriers to care. CRPs were recruited through the NRSP district support office and the lay community women in both groups were, in turn, identified and recruited through the CRPs through a mix of convenience and snowball sampling. Owing to the strong connections and linkages in the community, all the women who were reached out by CRPs from NRSP agreed voluntarily to participate in the study.

There were 12 women in each of these three groups (36 women in total), and a total of six FGDs using semi-structured questionnaires were conducted, two within each of the three groups of women. The FGDs with the CRPs were carried out at the NRSP office whereas the discussions with the lay community women were carried out in the houses of CRPs. Each FGD lasted approximately one hour. Discussions covered household decision-making and family dynamics; perceived gender norms in the community; the concept of health and common health issues for women; inequities of existing healthcare system; knowledge, attitudes and perceptions around TB; and the adequacy of local healthcare system for TB treatment. CRPs were also asked about their opinions on the need for a health program that would focus on women in Tando Allahyar and their motivation for being involved in such a program as volunteers.

### 2.3. Data Management and Analysis

All the FGDs were conducted by a trained researcher, experienced in moderating focus groups discussions in a variety of settings. The FGDs were audio-recorded and transcribed in the local language by two research associates, who were both skilled in transcription. Transcripts were then translated into English language for the purpose of analysis. Data were stored electronically in password-protected and encrypted computers. Data was manually analyzed with deductive analysis based on the SEM. Additional inductive analysis using a grounded approach was used to ascertain other emerging themes and sub-themes during the process of transcription and analysis. The coding framework was refined though a series of meetings between the researchers as it was being developed.

### 2.4. Ethical Considerations

Ethical approval for the study (Reference IRD_IRB_2020_03_007) was obtained from the Institutional Review Board (IRB) of Interactive Research & Development, registered with the Department of Health and Human Services, USA. Participation was voluntary, and all participants were informed about their right to withdraw at any time, or to refuse to answer any questions. Verbal consent was obtained from all participants, as was appropriate in a context of low rates of literacy, and they were ensured of strict confidentiality. Women were transported to the study site by vehicles owned by NRSP. Refreshments were provided but no remuneration was provided to the participants. Research was discussed with and approved by the authorities from Provincial Tuberculosis Programme and community leaders from NRSP prior to FGD initiation.

## 3. Results

Interpretation of the results that follow should be considered in light of the participants’ characteristics. Marriage and children were among the reported characteristics, as in Pakistan these are important markers of a woman’s stage of life which may be a determinant of her views. A total of 36 women were interviewed. The majority (*n* = 29) of the women were married with seven unmarried at the time of the FGD, and none previously married. Most (*n* = 27) of the married women were mothers, with the number of children ranging from one to nine, and one participant was pregnant for the first time. Among the lay community women, a majority (70%) of the participants were working as laborers. A few participants also reported as having independent sources of income through cosmetic and sartorial work.

### 3.1. Individual Level Analysis

General health awareness and health seeking:

Health was defined by participants as the absence of illness and linked it with diet, hygiene, and use of medicines for illness. The common health problems identified were hepatitis, diabetes, hypertension, diarrhea, TB and pneumonia.

“*There is sugar and blood pressure. If someone with blood pressure issues consumes sugar, then they immediately become diabetic. Besides that, there is fever, cough, TB; these problems have become more prevalent*.”

—Lay community woman, 26 to 49 years group

Participants identified anemia, diabetes, urinary tract infections (UTIs), menstrual cramps and TB as problems particularly facing women. Furthermore, participants also reported a recent increase in incidences of neonatal mortality, though reasons for this were not suggested.

“*Many women lose their first child. Often the new-born’s heart doesn’t work or stops working after a few days*.”

—Lay community woman, 18 to 25 years group

TB was understood to be a contagious disease by the study participants and also described it as “common”, easily transmissible, and treatable. Nearly all participants either knew, or knew of, someone affected by TB, currently or previously.

“*TB is a common disease and it is curable. I’ve heard when one person has TB in the household then it spreads to the entire family. If the person has children, then the children may also get it*.”

—Community Resource Person

“*My mother had TB and then my three-year old daughter caught it as well. Now they are completely fine after completion of treatment*.”

—Community Resource Person

All participants were generally aware of the common symptoms of TB and identified persistent cough, bloody cough, breathing difficulty, fatigue, fever, and weight loss as being associated with TB. While some participants were able to identify bacteria as the cause of TB and were also knowledgeable about airborne transmission, more than half of the participants believed that TB could be caused by drinking cold drinks, eating oily foods, smoke and dust, and sexual intercourse. In terms of diagnosis, participants largely spoke of clinical evaluation by a doctor’s consultation and did not exhibit knowledge of the diagnostic tests that may be required. Participants reported being aware of a treatment “course” for TB, with some participants referring to a six-month time span. At the same time, participants reported first using traditional remedies (“*totkay*”) such as use of locally available herbs, self-medication, or going to local *hakims* for medical care. Going to the doctor in the city was a resort after these methods had failed, generally when the cough persisted for over a month, or other symptoms such as fever or haemoptysis developed. Discussions centered on pulmonary TB and participants did not report any perceived difference in symptoms between men and women.

Overall, the CPRs demonstrated better knowledge and awareness about TB, its cause, prevention, and treatment, likely due to their experience as frontline workers in public health-related projects.

### 3.2. Interpersonal Level Analysis

Highly patriarchal societal norms were described as infusing all aspects of life. Participants reported that major decisions such as expenditure on housing, children’s education and healthcare were controlled by men. Income earned by women themselves was also reported to be managed by their husbands. Women’s decision-making was found to be limited to routine household management such as cooking meals, a realm where men were not reported to be involved. Experiences of greater female autonomy was only reported for households in exceptional circumstances whereby the husband was medically incapacitated.

“*My husband says that children should be engaged in active labour work and earn money while I say that they should go to school*.”

—Lay community woman, 18 to 25 years group

Men were perceived as the ones who were prioritized for receiving education, responsible for financially supporting the household, and responsible for performing physical labor. Spending for young boys was also considered more rewarding as they are believed to provide for the family as they grow up and become financially active. Girls were described to face neglect and not considered worthy of healthcare expense because they eventually leave the family as a result of marriage.

Other barriers to TB care access reported for women were the same as women experience for general healthcare. The demands of household chores meant that women cannot rest or travel to seek care. Permission and accompaniment from a male partner or household member are required for women to travel, and this is not always provided, and women cannot necessarily cover the costs of the required journey.

“*Men can stay alone in the hospital while there should be someone with the woman. When a woman goes to the hospital, her husband or brother goes with her. When the breadwinner of the house goes with the woman then everyone in the family gets worried. There are all kinds of resources in Kotri, but because of these reasons, women don’t go*.”

—Lay community woman, 26 to 49 years group

“*If we need to travel to the city for any purpose, we must seek our husband’s permission and travel with a male family member. If the husband does not give us permission, we do not go*”

—Lay community woman, 26 to 49 years group

Similarly, women also felt that they could not seek care due to social responsibilities as they were the primary care takers for households, children and the elderly. It was also reported that their health was often deprioritized over health of their children, husbands, or brothers.

“*If the son falls ill, he is sent to the hospital immediately but if the girl falls ill, she is told to take a tablet to make it better*.”

—Lay community woman, 26 to 49 years group

As working women, the CRPs reportedly exercised relatively greater freedom with regards to their expenses, mobility, and personal decision-making compared to lay community women. However, they too reported depending on their husbands and elder male figures of the family for long-distance travelling, larger expenses, and high-impact decisions related to work, household, children and healthcare.

“*If there is a big issue, for example, if there is a need to travel to Hyderabad or Karachi due to sickness, we obviously cannot travel without our husband or brother-in-law. We are not independent in doing such big things, but can take care of smaller issues ourselves*.”

—Community Resource Person

### 3.3. Community Level Analysis

Women’s social functioning was largely restricted to the household, the fields, and tending to children. However, these restrictions were found to reduce with age, with younger women facing a much more restrictive environment than older women.

“*You know that ‘maahol” (environment) is not suitable for girls that’s why we do not send them out of the house unless needed. Small kids roam in the streets all day, but we do not send our daughters outside as they grow slightly older*”

—Lay community woman, 26 to 49 years group

Participants identified and recognized the value of education and vocational skills in gaining financial autonomy and in turn attain larger control over their health and well-being.

“*If a woman has vocational skills then she will never have to beg anyone for money*.”

—Lay community woman, 26 to 49 years group

“*Women who are “khud-mukhtaar” (self-reliant) can take better care of their own health and well-being. They buy groceries and food items for the house and hence are free to consume those whichever way they like*”

—Lay community woman, 18 to 25 years group

Participants were also cognizant of the challenges that women face in attaining education and skills. However, with the increasing inflation and economic hardship it was reported that some men are more accepting of women taking jobs for remuneration.

### 3.4. Environmental/Structural Level Analysis

A key barrier to health services emphasized in all focus groups was the lack of medical facilities in the village. The existing structure of healthcare system was described as inadequate to deal with the reported health challenges at the village-level. Participants reported that people travel to nearby cities including Kotri (50 km), Hyderabad (40 km) and Karachi (200 km), to seek testing and treatment for TB since there was no clinic or doctor available in the village. The lack of facilities was also linked to poor individual-level knowledge about TB and delayed treatment seeking.

Transportation was a major limitation as public buses were either not available or delayed. Even when public transport was accessible and available, due to long routes and frequent stops it was challenging to reach the city for treatment and many patients were described as having suffered critically during the journey. Private transport was expensive and scarcely available. In addition, these challenges were further exacerbated for women due to their inability to travel alone and their lack of financial autonomy.

Individuals’ experiences in the hospital or clinic were reportedly highly dependent on the patients’ socioeconomic class. At a village level, when medicines are available, they are reportedly provided only to influential individuals, who have either a resource to offer as bribes or are connected to key resource persons at the health facility or the government. Impoverished patients were reportedly often neglected even if they have a *sehat* voucher (a coupon or a pass given to underprivileged households by the local government that allows them to avail healthcare services free of cost from public hospitals.) Moreover, participants preferred visiting private facilities as it was easier to receive treatment compared to a public facility where doctors and staff were often inattentive, and it was difficult to receive services without references.

“*Everything is done on referential requests [bribes]; if you have a reference then you immediately get the treatment or else you are made to wait and often the doctor leaves and you have to come again. Therefore, we don’t go to Civil (Hospital) and prefer to go to private facilities*.”

—Lay community woman, 18 to 25 years group

To counter barriers faced by women, participants suggested that a well-equipped health facility with quality service delivery at a reasonable distance from the villages around the city of Tando Allahyar should be established.

They further highlighted that the healthcare providers at these facilities should be empathetic, free of prejudice, and committed to treating patients without any monetary incentive. Social injunctions on contact between men and women inhibit women seeking care from male healthcare providers and few participants suggested the idea of having female healthcare providers locally referred to as “lady doctors” at these facilities. Some participants also proposed the idea of financial aid for patients belonging to lower socio-economic status; this financial aid would include coverage for treatment and transportation to and from the health facility.

CRPs had similar concerns regarding access to healthcare as that of lay community women. They stressed the need for affordable and reliable health services and facilities in Tando Allahyar at a reasonable distance from the surrounding villages. The participants strongly advocated for the need of household and community-level health awareness campaigns.

“*How can they go to the doctor; transport is an issue, cost of treatment is an issue, having small kids to take care of at home is an issue*…”

—Community Resource Person

## 4. Discussion

At an individual level our findings about barriers to TB care access in the Sindh Province reflect those noted about healthcare in general for women in Pakistan [29,30]. Aligned with Khan and colleagues [31], we noted that at an individual level biomedical TB treatment seeking was inhibited by limited knowledge and initial use of traditional healthcare systems. Like Shaikh and colleagues [32], we noted that the roles of care played by women and the demands of household chores undermined their ability to leave the household space to seek care. We echo others in noting that the costs of care, limited finances, and women’s lack of independent income all inhibit care access [14,33], and in finding that the household prioritization of men and boys, and women’s reliance on male-decision-making undermines women’s health autonomy [32,34].

At a community level, we noted that the inter-personal norms are very largely representative of socio-cultural norms in the region. In addition, the lack of local healthcare facilities, and prohibitions on women—particularly young women being independent or moving about independently—all hamper treatment access. Finally, the difficulty attaining the skills that would allow for independent income generation also pose a barrier to women’s ability to change their dependent situations.

At an environmental/structural level, the distances women must travel to access health services coupled with the lack of available, inexpensive transportation makes accessing biomedical care difficult. The poor quality of public services encourages women to seek care in private healthcare facilities, where TB diagnosis is less likely, and the testing and treatment is expensive. Along with others [34,35], we note that in the context of the social prohibition on men and women spending time together, the lack of female healthcare providers undermines women’s care access. Importantly, we further found care access depends on social standing, the utility of the health vouchers under different social protection schemes is limited, and the use of bribes is ubiquitous. Given that bribing is a social practice that occurs between men, this may also be suggesting an additional (and very largely unspoken) way in which women do not control their own healthcare decisions and access.

The structure of our research was locally unique in that our inclusion, and differentiation, of lay people and CRPs showed that even limited engagement in community work made a difference not only to health knowledge, but also in women’s self-efficacy and their ability to attain charge of their life and health decisions. Our separation of age-cohorts, where age was used as a proxy for life-stage, further suggested that gender plays a greater limiting role on younger and unmarried women than it does on older women. This phenomenon is particularly common in the uptake of maternal and child healthcare A previous study from Pakistan reports that most decisions regarding uptake of antenatal healthcare services during pregnancy are taken by the husband, or older women like a mother-in law [36]. Other examples for barriers to access of healthcare for younger females in LMICs include lack of access to contraception and safe abortions for adolescents [37,38], limited access to awareness programs for young (child-bearing age) women regarding their own health and prevalent conditions [39] and restricted mobility to reach health care services [40]. In this study, we found that younger women may need additional support for gaining TB knowledge and accessing TB services.

Our study, to our surprise, found very low levels of TB-related stigma reported. This may be because the women in the focus groups had generally not been affected by TB themselves, but we have no reason to doubt the veracity of this claim, which was repeated across the three separate focus group discussions.

A range of facilitators to care access also emerged from our work. Already present facilitators included a reported lack of TB-related stigma, some (though limited) knowledge about TB infection and disease, and a broad-based understanding of tuberculosis as a curable disease. Facilitators that were suggested to improve healthcare access included health facilities closer to the villages and the availability of services of a higher quality. Improved services would have healthcare providers that demonstrate empathy, and serve everyone in need equally, irrespective of social standing and ability to pay bribes. It is notable that all the changes mentioned by participants that would facilitate care access were at an environmental/structural level, but no changes were suggested at a community or individual level. This may suggest that women do not see possibilities for change at a personal or community level and that basic gender equity awareness and advocacy will likely be a starting point of any women’s empowerment work.

The United Nations (UN) recognizes that addressing gender equality is crucial for social change. This is cate-gorically recognized in Sustainable Development Goals, which encourages a move towards greater equality of access to resources, community and household roles and lifestyles between women and men around the world (United Nations, NYC, NY, USA, 2015). Global evidence suggests that gender equality in the access to resources such as education, income and political representation has a positive effect on the health of females, particularly in LMICs [41]. To further the gender equality agenda for greater health outcomes, women, particularly in rural areas, must be sensitized to their rights in a contextually appropriate way.

While we have presented these barriers and facilitators as set in particular SEM realms, we note that each realm impacts the other. As others [42,43] have noted, patriarchal social norms prevalent in the Pakistani society at large, especially in rural areas, shape healthcare access. We found this at all levels. For example, individual level barriers (such as lack of individual access to finances), are shaped by community norms in which women do not earn an independent income. Environmental/structural barriers, such as lack of female doctors, are in turn, shaped by the norm that women do not work independently. Despite this interplay, we believe that the conceptual separation is useful, for it highlights entry points for interventions targeting change processes. To reach long term TB control goals, there is a strong need to address proximate risk factors and upstream determinants of TB [44]. The upstream determinants identified in this study include weak health systems, poor access and inappropriate health seeking behavior.

This research further validated our approach to TB programming which was tailored to be gender sensitive in terms of service delivery mechanism, service providers, and the target population.

Additional areas which could be tackled to improve women’s access to TB care include interventions that seek to shift the gender relation norms that undermine women’s decision making and autonomy. Microfinance and vocational training may serve as potential approaches to increasing women’s ability to generate income and secure livelihoods. The *Benazir Income Support Programme* (BISP), under which females from households identified below the poverty threshold receive unconditional cash transfers from the government, has shown to contribute to women’s empowerment [45]. Further research is needed to assess its impact on health outcomes, including for TB.

## 5. Limitations

The study was carried out in a single rural district in Sindh and may not be generalizable to urban parts of the country and rural parts of other provinces where there may be cultural differences. Furthermore, the interviews were conducted only among a limited number of women, none of whom were affected by TB themselves; therefore, the study did not capture in-depth information about men’s perspectives on barriers to healthcare for women, or the specific barriers to TB care. Another limitation is the lack of detailed baseline demographic variables as a tool for collecting some form of quantitative data. The semi-structured questionnaire was aimed to qualitatively analyze the general understanding of TB and access to healthcare for women in Tando Allahyar. Despite the wide reach of NRSP linkages in the community via referrals, the widely used snowball sampling method applied in this study has a known bias towards inclusion of those who have strong interrelationships or social connections [46]. The data collection method may also create a bias (group bias) where the opinion of one respondent may influence others, which is common in focused group discussions. The data collector made due efforts to keep the discussion open and encouraged participants to give their own truthful responses.

## 6. Conclusions

National TB programs should be informed by and respond to the ways in which gender effects individual, household and community practices that may increase the risk of TB and influence health seeking behavior. Gender roles and specific needs will have to be considered when designing and implementing these programs to ensure that health systems serve to address gender inequalities and advance health outcomes equitably [47]. Pakistan is unusual in that the M:F TB burden ration, at 1.1, is one of the lowest of any countries. Our paper has shown that a contributor to this may be the multiple layers of barriers women face accessing healthcare. We further showed that young women, who locally demonstrate a higher TB burden than men of the same cohort, were reported to experience greater challenges in TB care access than older women. This demonstrates a need for additional focus on younger women. This should include work that not only focused on environmental/structural changes, but also that focuses on women’s empowerment at the individual and community level, for ultimately, it is these changes that will lead to systemic improvement that goes beyond the successful treatment of TB and generates shifts towards a more gender-equitable world.

In showing that current systems that seek to support marginalized people to access healthcare, such as social protection schemes, may be critical, we are also revealing that we currently are failing to do enough. Our work shows that more needs to be done to counter the class-related stigma as well as additional research conducted into how this can be successfully implemented. At the same time, our work confirms much of what has already been demonstrated in Pakistan. This suggests that while a gender-analysis should ideally be undertaken before any project design is set out, where this is not possible, program implementers can, as we did, shift towards improved gender-responsive programming by drawing on the available literature.

## Data Availability

The dataset used for analysis during the current study is available from the corresponding author on reasonable request.
